# One-Step Laser-Guided Fabrication of 3D Self-Assembled Graphene Micro-Rolls

**DOI:** 10.1021/acsnano.4c17646

**Published:** 2025-02-03

**Authors:** Yi Chen, Xupeng Lu, Ganggang Ma, Minseong Kim, Ruohan Yu, Haosong Zhong, Yee Him Timothy Chan, Min Tan, Yang Liu, Mitch Guijun Li

**Affiliations:** † Center for Smart Manufacturing, Division of Integrative Systems and Design, The Hong Kong University of Science and Technology, Clear Water Bay, Kowloon 999077, Hong Kong SAR, China; ‡ State Key Laboratory of Advanced Displays and Optoelectronics Technologies, The Hong Kong University of Science and Technology, Clear Water Bay, Kowloon 999077, Hong Kong SAR, China; § State Key Laboratory of Advanced Technology for Materials Synthesis and Processing, Wuhan University of Technology, Wuhan 430070, China; ∥ Wuhan University of Technology, The Sanya Science and Education Innovation Park, Sanya 572000, China; ⊥ Department of Applied Physics, Hong Kong Polytechnic University, Kowloon 999077, Hong Kong SAR China

**Keywords:** laser-induced graphene, self-assembly, strain engineering, micro-roll, laser nanofabrication

## Abstract

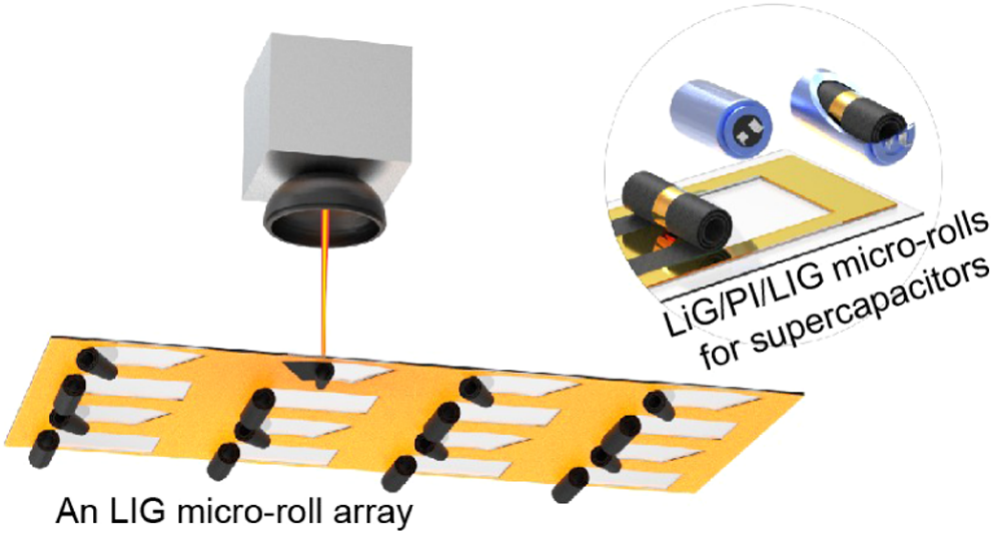

Laser-induced graphene (LIG) has been systematically investigated and employed because of the spartan laser synthesis and functional three dimensional (3D) foam-like structures. However, thermally induced deformation during laser processing is generally undesirable and, therefore, strictly suppressed. This work introduces a novel laser-guided self-assembly approach integrated into the fabrication of LIG to generate multiscale 3D graphene foam structures in a single step. Leveraging the photothermal effects of laser ablation on polyimide films, we achieve concurrent LIG production and self-assembly, enabling the transformation of two dimensional films into 3D micro-rolls. The process is finely tuned through interface modification and optimized laser parameters, allowing precise control over the geometry of the resulting structures. Systematic investigations reveal that varying laser power and line spacing effectively adjust the diameters of the LIG micro-rolls. Characterization indicates that the LIG micro-rolls can be fabricated with very large curvature and limited internal space, enhancing the potential for microscale applications. Furthermore, our laser strategy facilitates the creation of symmetric, asymmetric, and double-tube micro-rolls, underscoring its design flexibility. This work highlights the potential of the laser-guided self-assembly strategy in graphene nanomaterials and miniaturized applications, which has been exemplarily verified through the LIG micro-roll supercapacitors.

## Introduction

Graphene-related nanomaterials have been thoroughly investigated, demonstrating excellent chemical, mechanical, optical, and electrical properties.^1−3^ In contrast to single-layer or few-layer graphene, three-dimensional (3D) graphene structures, like graphene foam, graphene sponge, and graphene aerogel, offer distinct advantages for specific applications.^4^ Laser-induced graphene (LIG), also referred to as amorphous graphene or pseudographene, was a 3D graphene foam with a large specific surface area.^5−8^ The LIG material was initially generated through a straightforward laser ablation process on polyimide sheets in ambient air, and the target gradually expands to natural materials like woods and leaves.^9,10^ These properties make LIG a promising candidate for cost-effective and large-area production of 3D foam-like graphene. Additionally, LIG exhibits remarkable thermal and electrical conductivity along with high light absorption. Over the past decade, LIG has found widespread application in energy storage,^11−13^ electrocatalysis,^14,15^ environmental remediation,^16−19^ biotechnology,^20−22^ and various sensing systems.^23−26^

While the exact reaction mechanism remains a topic of ongoing research, the transformation of polyimide (PI) into LIG appears to involve photochemical, photothermal, or a combination of both processes, with the photothermal method being more likely to produce LIG when using infrared laser sources.^7,8^ The photothermal effect generates localized regions of extremely high temperature, facilitating the conversion of sp^3^ to sp^2^ carbon atoms, which has been extensively studied.^27,28^ This extreme temperature gradient can also induce substantial thermal stress, leading to significant deformation of the film. However, such thermal deformation is generally considered undesirable and irregular,^29^ and thus should be minimized. Currently, there is no systematic control over laser-induced deformation to create multiscale 3D LIG structures effectively.

Thin film self-assembly techniques are strain engineering methods that facilitate the intentional large-scale mechanical deformation of thin films, such as folding,^30^ buckling,^31^ and rolling,^32^ using internal or external forces. This mechanical deformation process transforms two dimensional (2D) flattened films into 3D microstructures, which have been applied as photodetectors,^33,34^ microbatteries,^35,36^ solar modulators,^37^ etc. Recently, we introduced a laser-guided self-assembly approach to create 3D micro-rolls from metal thin films.^38^ With proper interfacial modification and laser settings, this method allows for pattern definition and self-assembly of thin films in a single laser scribing step. It provides digital control over the self-assembly process, including parameters like area, direction, and curvature, due to the point-to-point interaction between the laser and the material, alongside the photothermal effect and subsequent film release. Additionally, this strategy has advantages such as being mask-free, suitable for large-scale production, and operable in ambient air.

In this work, we present a laser-guided self-assembly approach within the LIG fabrication process to create multiscale 3D graphene foam structures. LIG fabrication and self-assembly processes occur concurrently by employing interface modification, prepatterning, and optimized laser scanning parameters. This transient heating from the photothermal effect facilitates the conversion of polyimide (PI) films into LIG, allowing the thin films to release and roll up. In addition to mechanical deformation, three types of micro and submicron structures are formed on the LIG micro-rolls, which have been thoroughly examined and compiled. We have demonstrated that varying laser power and line spacing can effectively modify the geometry of the LIG micro-rolls without requiring any pretreatments or post-processing. With optimized laser parameter sets, the inner diameters of the LIG micro-rolls can reach nearly zero. The quality of LIG under different laser settings has been characterized. Moreover, diverse patterns, scanning paths, and sequences have been employed to produce symmetric micro-rolls, asymmetric micro-rolls, and double-tube micro-rolls, highlighting the extensive design flexibility of our approach. The single-step fabrication of LIG micro-rolls results in unique multiscale 3D graphene structures, hinting at exciting possibilities in compact devices like energy storage systems. Specifically, parametric LIG-PI-LIG micro-rolls have been fabricated as small-scale 3D supercapacitors, obtaining enhanced capacitive properties compared to the planar one. This instance indicates the superiority and potential of proposed LIG micro-rolls in various miniaturized applications.

## Results and Discussion

### Fabrication and Self-Assembly of LIG by Laser Scribing

**Figure 1:**
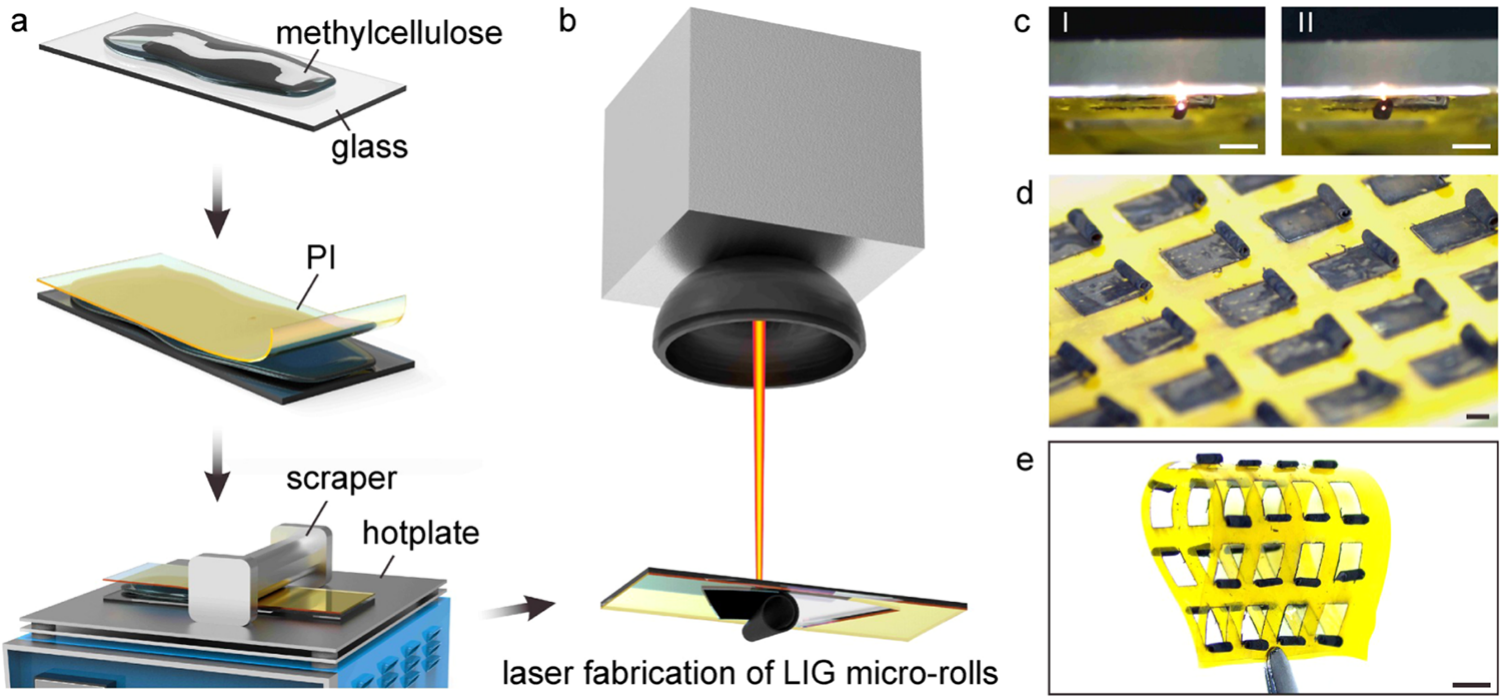
Laser fabrication of LIG micro-rolls. (a) The preprocesses include dropping the methylcellulose solution onto the glass substrate, attaching the PI thin film, and squeezing the PI film with a scraper. (b) The laser writing from the backside of the glass, during which the LIG forms and rolls up. (c) Two captures showing a real LIG microroll during laser scribing. Scale bar, 1 mm. (d) A fabricated LIG microroll array. Scale bar, 1 mm. (e) A PI thin film with many LIG micro-rolls. Scale bar, 2 mm.

Figure 1a illustrates the pretreatment steps for preparing LIG micro-rolls. Initially, a methylcellulose solution was applied to a clean sodalime glass substrate. Subsequently, a 25 μm thick commercial polyimide (PI) film was affixed to the glass. The methylcellulose solution effectively wetted the PI film, filling the space between it and the substrate. The entire sample was then heated on a hot plate, causing the solution to evaporate and resulting in the formation of numerous bubbles beneath the PI film. These bubbles hindered the film’s adhesion and created an uneven surface. To eliminate these gas bubbles, a stainless-steel scraper was used, designed to create a 5 μm thick adhesion layer. This squeezing process was repeated every 5 min as new bubbles continued to form. After 20 min of heating and four rounds of squeezing, the PI film was securely bonded to the glass substrate via a layer of cured methylcellulose. Figure S1 shows the scanning electron microscopy (SEM) images for cutting and peeling PI film. The cured methylcellulose layer shows a weak adhesion to the PI film because the peeled PI is attached with minimal methylcellulose pieces.

The subsequent laser scribing step is depicted in Figure 1b. A commercial laser engraving machine operating at a wavelength of 1064 nm in continuous mode was employed, and the entire process was conducted in ambient air. Laser scribing was divided into two phases: pattern definition and the fabrication of LIG micro-rolls. First, a high fluence laser was used to ablate specific areas of the PI film, defining the desired patterns and isolating these sections from mechanical interactions. Next, a lower fluence laser was used to scan the patterned film, with the laser beam controlled by a galvanometer system. With the appropriate laser settings, the patterned PI film was converted to LIG during the scanning process. Concurrently, the methylcellulose interlayer was also burned due to heat conduction, allowing the thin film to detach from the substrate. As the laser scanned line by line, the PI film transformed into LIG and rolled up accordingly.

Figure 1c(I,II) presents video stills of the typical LIG fabrication and self-assembly processes, which are taken from Video S1. The laser energy was absorbed in the areas where the PI film burned, with both captures confirming that the laser beam’s position aligned with the micro-rolls, demonstrating the control over the LIG fabrication and self-assembly. Figure 1d displays an array of as-fabricated LIG micro-rolls, achieved with laser settings of a scanning speed of 400 mm s^–1^, a calibrated power of 1.24 W, and a line spacing of 2 μm. The interaction between the laser and PI film will be discussed in the following section. One side of the LIG micro-rolls could be securely attached to the PI films with the correct laser parameters, allowing the PI films with LIG micro-rolls to be removed from the substrates with suitable mechanical force. Figure 1e shows a PI film featuring multiple LIG micro-rolls being held with tweezers, highlighting the potential for flexible applications.

In addition, we have tried to simplify the pretreatment steps. According to the research by Li and coauthors,^39^ we use deionized water or ethanol instead of methylcellulose layer as the interlayer, and the results are shown in Figures S2 and S3. The process flow is the same as in the manuscript, except that deionized water or ethanol is used instead of methylcellulose solution. Here, the power range is set between 0.46 and 1.34 W, with a scanning speed of 400 mm s^–1^ and a line spacing of 2 μm. From 0.46 to 0.66 W, the PI film is gradually converted into LIG and begins to deform, but it can still maintain a continuous film morphology. Further increasing laser power, the LIG film shows obvious damage and irregular deformation. We think the irregular deformation is due to the low viscosity of water and ethanol, which causes the unexposed PI film to release from the substrate over a large area, as shown in Figure S3-VII, VIII, and IX. With the unstable boundary condition, regular deformation will be difficult to form. In addition, the large-area release of the PI film reduces the heat conduction efficiency, so heat accumulates in the film and tends to create ablation holes easily.

We have also tried using PI tape directly. Commercial PI tape (3 M Polyimide Film Tape 5413) is directly attached to a cleaned soda-lime glass. Then, the sample is laser scribed with a scanning speed of 400 mm s^–1^ and a line spacing of 2 μm, and the laser power range is set between 1.24 and 1.94 W. Figure S4 shows the typical result. From 1.24 to 1.44 W, the PI tape is firmly attached to the substrate. The PI tape begins to peel off at 1.54 W. Increasing laser power to 1.64 W, the PI film is completely released from the substrate and bent. Further increasing laser power to 1.74 W, the PI film shows irregular deformation and severe damage. Comparing the results of Figures S2–S4, we can find that the PI tape can only be peeled from the substrate at a relatively higher laser power, which is very likely due to the stronger interfacial adhesion hindering the release of PI. The stronger laser power should be responsible for the severe damage to the sample of Figure S4-VI. In addition, many bubbles appear underneath the PI tape, which should be trapped by the strong interfacial adhesion. Maybe we should try some less sticky PI tapes later.

### Mechanism for the LIG Fabrication and Self-Assembly

**Figure 2:**
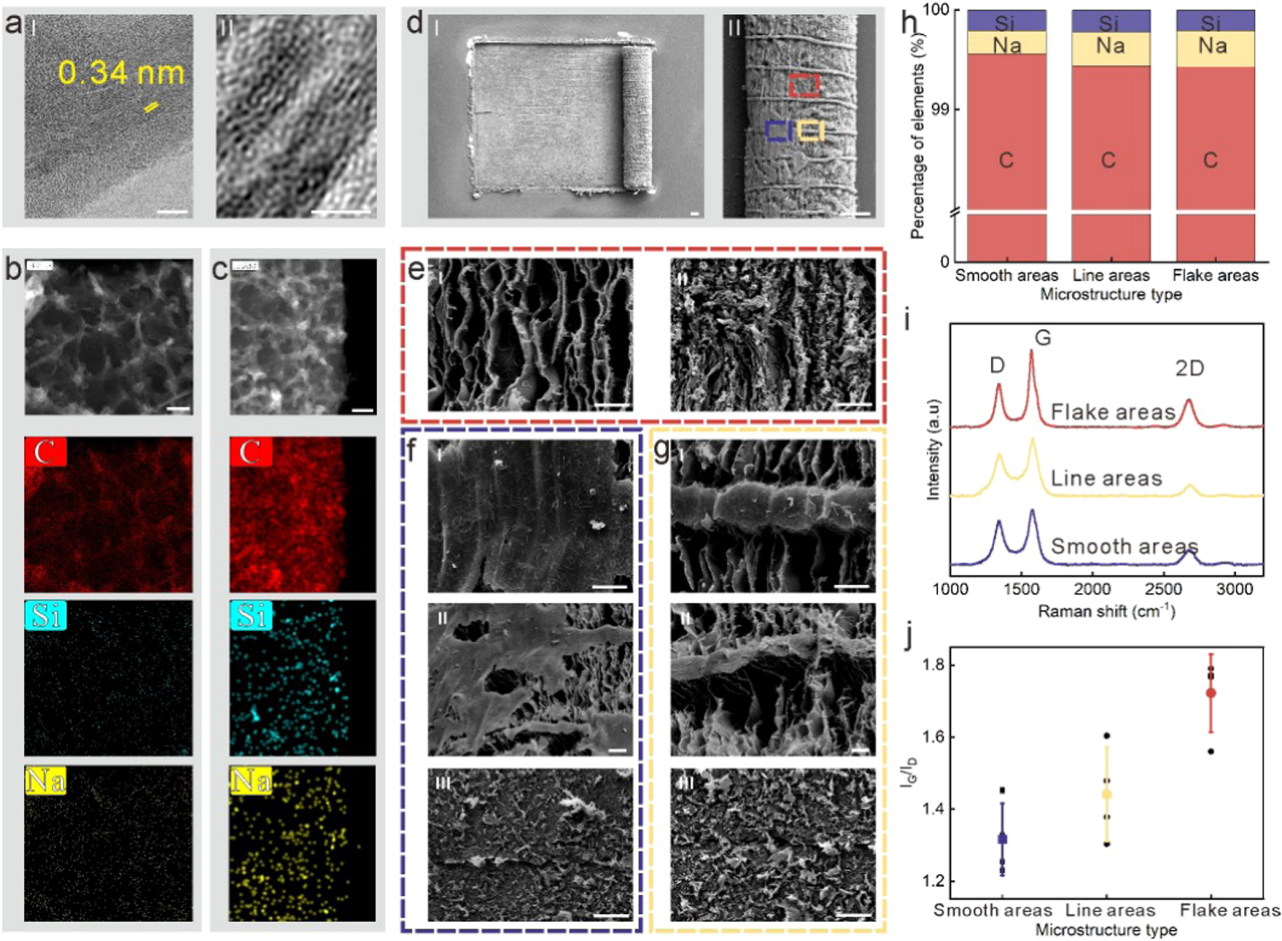
Nano/microscale structures on LIG micro-rolls. (a) A TEM image of a LIG flake edge (I), and a STEM image of the grain boundaries. Scale bar, 5 nm. (b) A TEM image of the porous structures on a LIG flake, and the corresponding mapping results showing the elements C, Si, and Na. Scale bar, 100 nm. (c) A TEM image of a curved LIG flake and the corresponding mapping results showing the elements C, Si, and Na. Scale bar, 50 nm. (d) A SEM image of a LIG microroll (I) and its amplified area (II). Scale bar, 100 μm. (e) The flake structure on the LIG microroll surface (I) and the corresponding substrate surface (II). Scale bar, 10 μm. (f) The smooth structure on the LIG microroll surface (I), the tilted view of the smooth structure (55°) (II), and the corresponding substrate surface (III). Scale bar, 10 μm. (g) The line structure on the LIG microroll surface (I), the tilted view of the smooth structure (55°) (II), and the corresponding substrate surface. Scale bar, 10 μm. (h) The atomic percentage of elements for all three types of structures. (i) The representative Raman curves for three types of structures. (j) The statistic of *I*_G_/*I*_D_ values.

The transformation of polyimide (PI) into LIG using a 1064 nm laser has been reported.^19,40−42^ The localized interaction between the laser and the PI film leads to transient heating, creating the high-pressure and high-temperature conditions essential for graphene formation. Figure 2a-I shows a transmission electron microscopy (TEM) image of a flake from a LIG microroll. The lattice spacing was measured to be about 0.34 nm, matching the value in published research.^5,43^Figure 2a-II shows grain boundaries from the same LIG flake. Figure 2b shows a typical porous structure on a LIG flake. Figure 2c shows a curved LIG flake. Their corresponding energy-dispersive X-ray (EDX) mapping results show the existence of only three elemental components: carbon, silicon, and sodium, which are distributed quite uniformly. The carbon likely originates from the PI film, whereas the silicon and sodium appear to be transferred from the soda-lime glass to the LIG microroll surface. Notably, no oxygen was detected, indicating that the LIG has not undergone significant oxidation. Carbon atoms account for more than 97% of the LIG flakes.

The interaction between the laser and PI also causes changes in the micro- and submicron-scale morphology of the LIG microroll surface. Figure 2d-I is a SEM image of a typical LIG microroll. Figure 2d-II provides a magnified view of a specific area, which shows three structural types: flake structures (the red dashed box), smooth areas (the blue dashed box), and line areas (the yellow dashed box). Figure 2e-I offers a closer look at the flake structures, revealing distinct gaps between them. According to existing research,^5^ these gaps form through two steps: (1) the creation of porous structures as gases (like CO and CO_2_) escape from the PI film during laser processing; (2) the merging of these pores as additional energy is applied to burn the edges. These flake structures extend radially along the micro-rolls, which aligns with the direction of the laser scanning. Consequently, their orientation is highly consistent. Figure 2e-II shows the substrate’s corresponding area, which exhibits many flake structures with the same radial orientation. The glass substrate is not visible in Figure 2e-II, indicating that the peeling-off position likely occurs within the PI film.

Figure 2f-I presents a magnified view of a smooth, bright area that is flat and covered with a significant amount of polymer nanofibers, which seem to be produced by thermal decomposition of methylcellulose. Figure 2f-II offers a tilted perspective of the same location, revealing the flake structures beneath this flattened surface. This flattened region is likely the bottom surface of the PI film that adheres to the substrate, further confirming that the flake structures are embedded within the PI film. In Figure 2f-III, the corresponding area on the substrate shows fragments of carbonized PI attached to the glass surface. This suggests that the peeling-off position is at or very near the bottom surface of the PI film.

Figure 2g-I provides a close-up of a typical line on the LIG microroll. This narrow, smooth line, which also has a considerable amount of nanofibers, resembles the flattened surface described earlier. Figure 2g-II shows a tilted view of the same line, again revealing the flake structures beneath it. These line features likely correspond to the surface of the PI film that is in contact with the substrate. Comparing Figure 2g-I,III, we observe that the line structures correlate with crack patterns on the glass substrate. The cracks in the glass are likely a result of the high temperature and pressure involved. Figure 2g-III displays several carbonized PI fragments adhering to the fractured glass surface. The broken section of the glass (central area) aligns with the line area on the microroll. It is suspected that these cracks facilitated the detachment of the PI, resulting in the smooth line areas being coated with methylcellulose, while the surrounding undamaged regions remained firmly adhered to the PI film, causing the adjacent PI to separate from the interior.

The EDX mapping results for the three structural types are depicted in Figure S5. The results are consistent with the TEM EDX mapping. All structures reveal only three elemental components: carbon, silicon, and sodium, distributed quite uniformly. Figure 2f illustrates the atomic percentages of the elements present. Carbon constitutes over 99.4 atomic percent across all structures, which aligns with our expectations for laser-induced hydrocarbon synthesis. Silicon represents 0.21 atomic percent, while sodium accounts for 0.23, 0.34, and 0.37 atomic percent in the smooth, line, and flake structures, respectively. Given that Figure S5c of line structures contains both smooth and flake areas, it makes sense that the sodium content in the line structures falls between those of the smooth and flake structures.

X-ray photoelectron spectroscopy (XPS) characterization has been done for LIG micro-rolls. Figure S6 shows a typical XPS result. The corresponding laser settings include a laser power of 1.03 W, a scanning speed of 400 mm s^–1^, and a line spacing of 2 μm. Figure S6a shows oxygen and nitrogen elements besides carbon, silicon, and sodium, meaning oxygen and nitrogen content on the LIG surface is much higher than in the deep area. Figure S6b shows sodium’s binding energy of 1072 eV, indicating a +1-valent chemical state. Figure S6c shows silicon’s binding energy of 102 and 104 eV, indicating a +4 or 0-valent chemical state. Because soda-lime glass only contains +4 or +2-valent silicon, PI and methylcellulose interlayer do not contain silicon, so the silicon in LIG is likely to be in a +4-valent chemical state.

We have also tried another type of glass, fused silica, to replace the soda-lime glass because fused silica does not contain sodium elements. Figure S7 shows a typical LIG microroll on a fused silica substrate. The sample is fabricated with a laser power of 1.03 W, a scanning speed of 400 mm s^–1^, and a line spacing of 2 μm. Figure S7a,b are the top view and titled view of the LIG microroll, and Figure S7c shows an amplified area of the microroll surface. Comparing this LIG microroll to that in Figure 2, we find this LIG microroll shows only one type of micro- and submicron-scale morphology: flake structure. And there is no obvious cracking on the fused silica substrate, which seems reasonable because fused silica has a higher softening temperature and a lower coefficient of thermal expansion than soda-lime glass. This result further confirms that the line structure and smooth structure in Figure 2 are related to the broken soda-lime glass surface. Figure S7d is the EDX mapping area. Only two elements, carbon and silicon, are found, which are shown in Figure S7e,f, respectively. The atomic percentages of carbon and silicon are 98.33 and 1.67%, respectively. The presence of silicon means that the substrate surface is still damaged, but it may be nanometer-scale. It shows that the LIG can be synthesized without Na, but it seems hard to do without Si. Perhaps we can try materials with higher thermal stability (like diamond film) as the substrate in the future.

The Raman spectroscopy results for the three structural types are presented in Figure 2i. All curves display the characteristic D, G, and 2D peaks, confirming the transformation of the PI film into LIG.^6^ The quality of the graphene can be assessed using the ratio *I*_G_/*I*_D_.^5^ We analyzed four regions for each structure type, as indicated by the black points in Figure 2j. The average *I*_G_/*I*_D_ values were found to be 1.32 (±0.10), 1.44 (±0.13), and 1.7 (±0.11) for the smooth, line, and flake structures, respectively. This analysis indicates that while the flake and smooth areas share similar elemental compositions, the flake structures exhibit superior graphene quality, meaning better crystallinity.

The driving force leading to the deformation is a synergistic effect due to the thermal expansion of the LIG/PI, the decomposition of the methylcellulose layer, and the possibly generated local high pressure. When the sample was exposed to the infrared laser of continuous wave mode, the PI layer absorbed the energy partially, leading to transient local hyperthermia and high pressure. The transient heat could result in the thermal expansion of the LIG/PI film. The high pressure promoted the release and deformation of the thin film, during which gas escaped from the free surface, resulting in porous formations. As the laser scanned, these pores merged to create elongated radial gaps. For most areas, the detachment occurred primarily within the LIG film since LIG is porous with low mechanical strength. Consequently, most of the microroll surface was composed of flake structures. For limited regions where adhesion was weaker, peeling occurred at the interface, leading to smooth areas. This weak interfacial adhesion may stem from the uneven distribution and size of gas bubbles beneath the PI film. Heat could be transferred to the methylcellulose layer, leading to the decomposition of the methylcellulose layer, and then many nanofibers were transferred to the LIG surface, especially on the smooth areas. Simultaneously, heat was transferred to the glass surface, causing it to fracture. As the glass surface was broken, the attached PI was likely released completely, resulting in numerous line features on the microroll surface. The presence of flake structures, smooth areas, and line features was observed in most LIG micro-rolls, with variations in the proportions of each structural type.

### Precise Control of the LIG Microroll Geometry

Researchers continually seek precise control over thin film self-assembly processes to fabricate 3D microstructures with specific curvature, area, orientation, and placement. A critical aspect of micro-rolls is their curvature diameters. Before the laser scribing step, the thin polyimide (PI) film was flattened and free from internal stresses. Consequently, the principal force responsible for rolling LIG films is likely the thermal stress and pressure generated by the photothermal effect. This allows for the diameter of LIG micro-rolls to be regulated through laser parameters. In this section, we focused on adjusting the diameter of LIG micro-rolls using two distinct laser settings.

**Figure 3:**
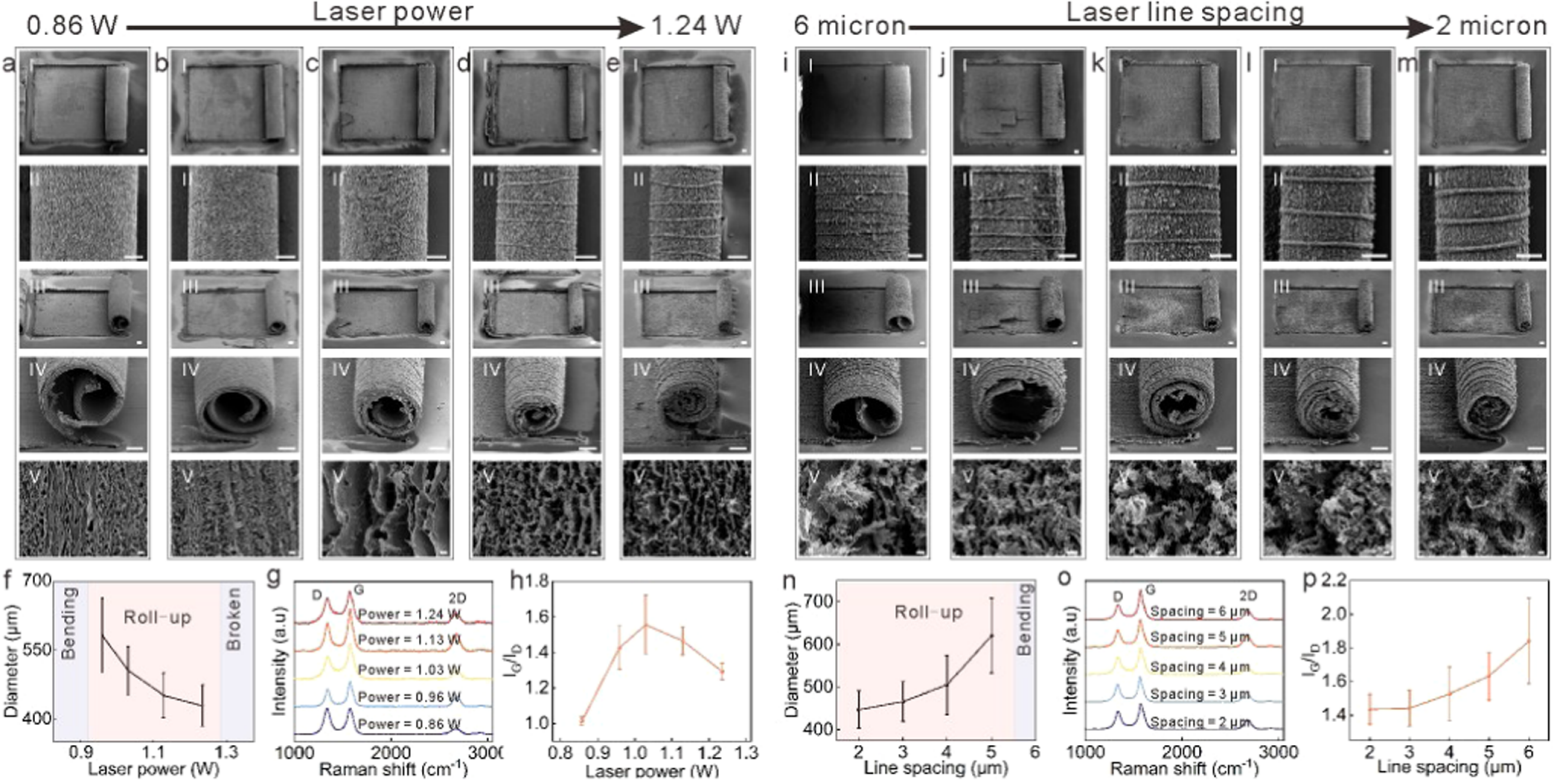
Adjustment of LIG microroll diameters with laser settings. (a–e) The micro- rolls with laser powers of 0.86, 0.96, 1.03, 1.13, and 1.24 W, respectively. (I) is the top view, and (II) is the amplified figure of (I). (III) is the tilted view (55°), and (IV) shows one end for every microroll. (V) is the micro/submicron-structures for LIG micro-rolls. (f) The statistic of LIG microroll diameters under different laser powers. (g) The representative Raman curves for LIG micro-rolls under different laser powers. (h) The statistic of *I*_G_/*I*_D_ values under different laser powers. (i–m) The micro-rolls with laser line spacing of 6, 5, 4, 3, and 2 μm, respectively. (I) is the top view, and (II) is the amplified figure of (I). (III) is the tilted view (55°), and (IV) shows one end for every microroll. (V) is the micro/submicron-structures for LIG micro-rolls. (n) The statistic of LIG microroll diameters under different laser line spacings. (o) The representative Raman curves for LIG micro-rolls under different laser line spacings. (p) The statistic of *I*_G_/*I*_D_ values under different laser line spacings. Scale bar, 100 μm.

Figure 3a–e illustrate the first method of diameter adjustment through varying laser powers, ranging from 0.86 to 1.24 W, while keeping other parameters constant: a laser scanning speed of 400 mm s^–1^ and a line spacing of 2 μm. The laser power was measured with a laser power meter, and the curves are presented in Figure S8. The patterned and rolled films measured 2.5 mm × 2.2 mm, all beginning the rolling process from the left short side. The calculated laser fluence varied between 107.1 and 154.4 J cm^–2^. We observed that the trend in LIG microroll diameters aligns with our previous findings. When the laser power was below 0.86 W, the thin film merely bent rather than forming micro-rolls. Conversely, at powers exceeding 1.24 W, significant ablation occurred, leading to broken and irregular micro-rolls, as Figure S9 shows. Further increasing the laser power results in severe damage to the LIG layer, preventing it from self-assembly.

Figure 3a–e(I,II) present the top view of the LIG micro-rolls, while Figure 3a–e(III,IV) depicts a tilted view along with one end of each microroll. As illustrated from Figure 3a–e, both the inner and outer diameters of the micro-rolls gradually decrease, accompanied by an increase in the number of layers. Notably, at a laser power of 1.24 W (Figure 3e-IV), the inner diameter approaches nearly zero, indicating that both the inner and outer diameters are very close to the theoretical minimum.

Figure 3f illustrates the relationship between the diameter of LIG micro-rolls and laser power within the operational window. We analyzed 12–15 samples for each power setting. As the laser power increased from 0.96 to 1.24 W, the diameter decreased from 582 (±80) to 428 (±45) μm. Notably, at powers below 0.86 W, the LIG films exhibited bending behavior, suggesting that larger curvature radii can be achieved. This implies that LIG micro-rolls with greater diameters could theoretically be produced by increasing the length of the precursor PI films.

In addition to diameter variations, different laser powers resulted in distinct micro- and submicron structures on the LIG micro-rolls. The microscopic surface morphology varied significantly with the laser power applied. At a laser power of 0.86 W (Figure 3a-II), the microroll presented a very smooth and intact surface. When the power was increased to 0.96 W (Figure 3b-II), the surface became less smooth, with the emergence of some linear features. Further increasing the power to 1.03 W (Figure 3c-II) revealed that most of the surface exhibited flake structures. At the same time, the linear areas became more pro-nounced, and the smooth regions diminished. At higher powers of 1.13 and 1.24 W, only flake structures and linear features were visible.

Figure 3a-V (at 0.86 W) shows that the apparently smooth surface contains evenly distributed submicron-sized pores, likely resulting from gas escaping during processing. Figure 3b-V (at 0.96 W) also displays submicron pores but with a greater density, which is reasonable as higher laser power promotes more gas release.^44^Figure 3c-V (at 1.03 W) reveals flake structures, indicating that the pores merge into elongated radial gaps during laser scanning. Figure 3d,e(V) depict smaller and denser flake structures, likely formed due to additional laser energy causing further ablation.

The typical Raman spectra of the LIG micro-rolls are depicted in Figure 3g. The D, G, and 2D peaks observed in all five micro-rolls further confirm the presence of graphene structures. To assess the quality of the LIG, we calculated the *I*_G_/*I*_D_ ratios for each laser power setting. The trend of *I*_G_/*I*_D_ relative to laser power (Figure 3h) aligns with findings from previous studies.^5^ The *I*_G_/*I*_D_ values rise between 0.86 and 1.03 W due to increased temperatures from higher power levels. However, as the power continues to rise, *I*_G_/*I*_D_ values decline, likely due to partial oxidation of the LIG.^15^

Figure 3i–m illustrate the second method of adjusting parameters by varying laser scanning spacings. The scanning spacing ranged from 2 to 6 μm, with a 1 μm interval between adjacent samples. Other laser settings were kept constant: a scanning speed of 400 mm s^–1^ and a power of 1.24 W. The patterned and rolled films measured 2.5 mm × 2.2 mm, starting the rolling process from the left short side. The calculated laser fluence varied from 154.4 to 51.5 J cm^–2^. When the laser scanning spacing was equal to or larger than 6 μm, the thin film tended to bend or roll into irregular shapes rather than forming complete micro-rolls. As the scanning spacing decreased, the diameter of the micro-rolls also reduced, indicating a tighter roll of the LIG film. Notably, at a line spacing of 2 μm (Figure 3m-IV), the inner diameter approached nearly zero, suggesting that both the inner and outer diameters were very close to the theoretical minimum, with approximately five layers present in the microroll.

Figure 3n illustrates the correlation between the diameter of LIG micro-rolls and laser line spacing within the operational window. We analyzed 12–15 samples for each spacing. The diameter increased from 438 (±38) to 620 (±87) μm as the laser line spacing rose from 2 to 5 μm. Importantly, LIG films exhibited bending at line spacings of 6 μm and above, indicating that larger curvature radii are possible. This suggests that LIG micro-rolls with greater diameters could be achieved theoretically by extending the length of the precursor PI films.

Figure 3i–m(I) present magnified images that showcase the microscopic surface morphology of the LIG micro-rolls depicted in Figure 3i–m(II). In contrast to the variations observed with different laser powers, the microscopic surface morphology of the LIG micro-rolls remains consistent across different laser scanning spacings. All micro-rolls exhibit linear features, with flake structures covering the majority of the surface, while smooth areas are absent.

As previously noted, varying laser power can lead to distinct micro- and submicron surface morphologies due to temperature differences. However, changing the laser line spacing behaves differently than altering laser power. Adjusting the laser power primarily modifies the spatial distribution of laser fluence while changing the line spacing involves both spatial and temporal modulation. The additional laser fluence is applied after a significant time interval, which minimizes the effects of heat accumulation. This results in similar peak temperatures for the various laser line spacings, likely explaining the minimal differences in their microscopic surface morphologies.

The primary distinction observed is that the surface-attached nanofibers increase markedly as the spacing decreases, as illustrated in Figure 3i–m(V). It appears that the nanofibers are transferred from the glass substrate to the surface of the micro-rolls during the laser process. Decreasing the laser line spacing leads to an increase in writing time, thus enhancing the duration of laser-induced forward transfer. This explains the more significant transfer of methylcellulose observed with reduced laser line spacings.

The typical Raman spectra of the LIG micro-rolls are presented in Figure 3o. The D, G, and 2D peaks observed in all five micro-rolls confirm the presence of graphene structures. We conducted a statistical analysis of the *I*_G_/*I*_D_ values for each line spacing, as depicted in Figure 3p. The trend of *I*_G_/*I*_D_ relative to laser line spacing differs from that associated with laser power. Within the range of 2–6 μm for laser line spacing, *I*_G_/*I*_D_ decreases as the spacing becomes smaller. The values for line spacings of 2 and 3 μm are comparable, indicating a stabilization of results. We hypothesize that this trend is linked to the oxidation of the LIG, as reduced line spacing results in more laser passes over the same area, allowing for gradual oxidation. At a line spacing of 3 μm, the LIG appears to be oxidized significantly, leading to stabilized *I*_G_/*I*_D_ values.

From this analysis, we conclude that both laser power and line spacing can effectively control the diameters of LIG micro-rolls without requiring pretreatments or postprocesses. Additionally, laser scribing produces distinct micro- and submicron structures on the surface of the LIG micro-rolls. The influence of laser power on these surface structures is more pronounced than that of line spacing. In terms of LIG quality, the interplay between graphene production and oxidation occurs when adjusting laser power, whereas oxidation becomes the dominant factor when varying laser line spacing.

### Precise Control of the Self-Assembly Behavior

The laser scanning path and sequence dictate the rolling direction, area, and positioning of the LIG thin film.^38^ This localized film release occurs as the laser scans the film line by line, allowing it to be released and rolled up accordingly. Below, we present various typical configurations of LIG micro-rolls.

**Figure 4:**
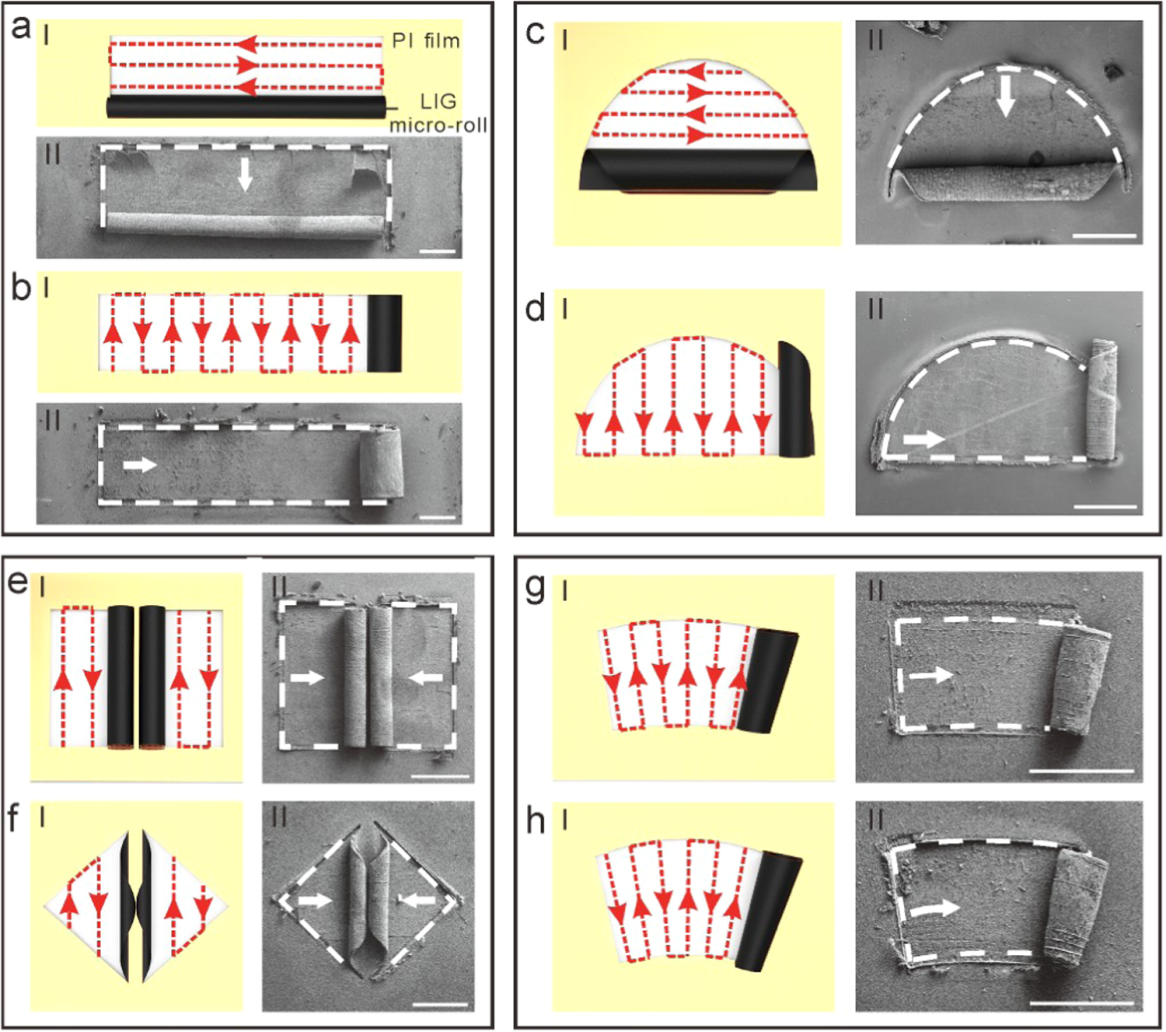
Control of rolling directions with laser scanning pathways. (a, b) The illustration of laser scanning pathways to make a rectangular thin film roll from the long sides and short sides (I) and real LIG micro-rolls (II). (c, d) The illustration of laser scanning pathways to make a semicircular thin film rolling from the two different directions (I) and real LIG micro-rolls (II). (e) The illustration of laser scanning pathways to make two rectangular thin films roll up (I), and real LIG double tube construction (II). (f) The illustration of laser scanning pathways to make two triangular thin films roll up (I), and real LIG double tube construction (II). (g, h) The illustration of laser scanning pathways to make asymmetric thin films roll up (I), and real LIG asymmetric micro-rolls (II). These red lines indicate the laser scanning paths and directions. These white dashed lines and white arrows indicate the boundaries of patterned films and rolling orientations. Scale bar (a–h), 1 mm.

In traditional thin film self-assembly methods, longer ribbons tend to roll from their longer side due to geometric effects. However, our laser technique enables rolling from both long and short sides. Figure 4a-I illustrates a long microroll formed from the long side of a ribbon, while Figure 4a-II shows a real long microroll with several layers. Figure 4b presents both an illustration and an actual sample of a short microroll rolled from the short side of the same ribbon, further demonstrating the effective control over rolling directions provided by the laser approach. Figure 4c depicts a microroll released from the arc boundary of a semicircular film, and Figure 4d shows one rolled up along the straight edge of the same semicircular film.

Additionally, we explore the combination of two micro-rolls. Figure 4e includes an illustration and a sample of double micro-rolls formed from two adjacent rectangular films, each rolling in opposite directions. Figure 4f similarly presents double micro-rolls from two adjacent triangular films, also with opposing rolling directions.

Beyond creating symmetric micro-rolls with uniform diameters, we also developed asymmetric micro-rolls using asymmetric precursor thin films and varied laser line spacings. The design principles are outlined in Figure S10. Figure 4g-I depicts an asymmetric precursor thin film in a sector shape, with laser scanning lines oriented vertically at the arc intersection points. The laser line spacing gradually decreases from the larger to the smaller arc. Figure 4g-II shows the actual roll-up of an asymmetric microroll featuring gradient diameters, with a primary arc length to minor arc length ratio of 24:23. Figure 4h-I presents another asymmetric precursor thin film, while Figure 4h-II showcases a real roll-up asymmetric microroll with gradient diameters, where the ratio of the larger arc length to the smaller arc length is 14:13.

In summary, both symmetric and asymmetric micro-rolls can be produced by carefully controlling laser scanning paths and sequences.

### LIG-PI-LIG Micro-rolls as Small-Scale Supercapacitors

**Figure 5:**
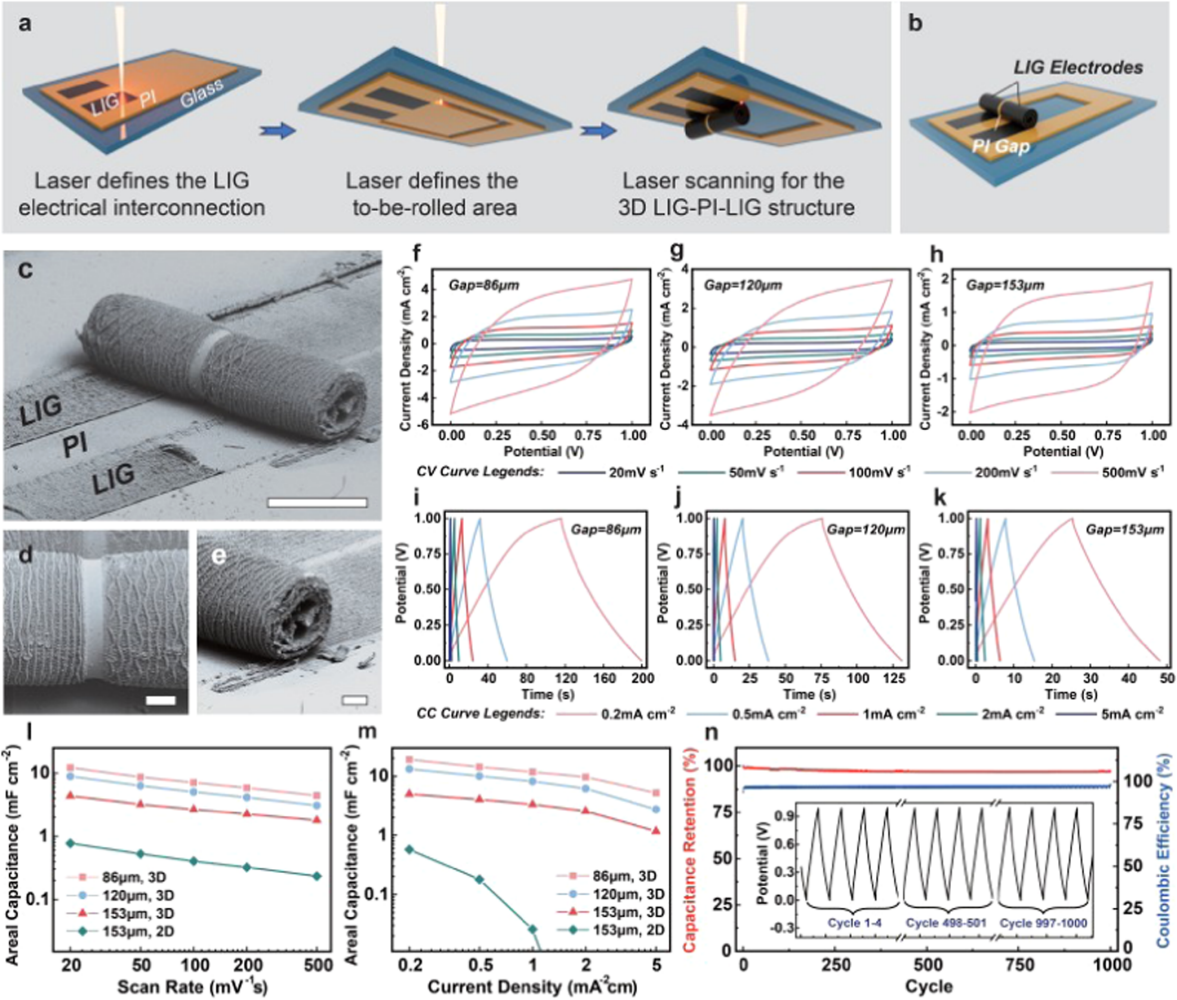
Supercapacitor application benefits from the 3D structures of the proposed LIG micro-rolls. (a) The fabrication process for the 3D LIG-PI-LIG structure based on laser manufacturing. (b) Schematic diagram of LIG-MRSCs. (c–e) SEM images of the overall device, PI gap, and LIG electrode. Scale bar (c), 1 mm. Scale bar (d, e) 200 μm. (f–h) CV curves of LIG-MRSCs with different gaps of 86, 120, and 153 μm across scan rates ranging from 20 to 500 mV s^–1^. (i–k) GCD curves of LIG-MRSCs with different gaps of 86, 120, and 153 μm across current densities ranging from 0.2 to 5 mA cm^–2^. (l) Areal capacitances of LIG-MRSCs gained from (f–h) in comparison to LIG-PPCs with a gap of 153 μm at the same CV scan rates. (m) Areal capacitances of LIG-MRSCs gained from (i–k) in comparison to LIG-PPCs with a gap of 153 μm at the same GCD current densities. (n) The cycling stability of the LIG-MRSC with a gap of 86 μm at the current density of 1 mA cm^–2^ during the 1000 GCD cycles, containing the GCD curves for cycles 1–4, 498–501, 997–1000.

LIG has been widely applied as flexible electrode materials in supercapacitors,^45−47^ batteries,^48−50^ electrochemical sensors,^51−53^ etc. With the laser-guided self-assembly strategy, the roll-up LIG can potentially increase energy density and reduce device size. Based on various LIG micro-rolls depicted in the previous sections, we propose a fabrication method for the 3D LIG-PI-LIG structure serving as LIG microroll supercapacitors (LIG-MRSCs). Figure 5a,b illustrate the whole process flow and architecture, which convert and roll up a precursor film area of 14.6 mm^2^ into a device with a projected area of 2.3 mm^2^ and a diameter of 700 μm. Two adjacent LIG electrodes and a PI separation gap are concurrently generated and rolled up according to the operational and intermittent regions of laser scanning (Figure S11a). As shown in Figure 5c–e, the LIG-MRSC sample features a smooth and seamless PI Gap. Its adjustable width is determined by the laser scanning path and spot size, showing a direct negative correlation with the capacitance. The LIG electrode surface exhibits regular flake and line structures, significantly increasing the actual electrode surface area while maintaining compact device dimensions. Notably, a stratified structure of LIG/PI/LIG/PI/··· spreads inward within the electrode cross-section of LIG-MRSCs, enabling the rolled LIG layers to be separated from each other. This phenomenon maximizes the surface area benefit of rolled-up electrodes, consequently leading to a substantial rise in the areal capacitance.

The electrochemical performance of the LIG-MRSCs was investigated with 1 M H_2_SO_4_ solution as the aqueous electrolyte via cyclic voltammetry (CV) and galvanostatic charge–discharge (GCD) tests. Figure 5f–h depict the CV results of three LIG-MRSC samples with PI separation gaps of 86 μm (Figure S12a), 120 μm (Figure S12b) and 153 μm (Figure S12c), respectively. The pseudorectangular shapes across scan rates of 20–500 mV s^–1^ suggest the effective electric double-layer capacitance (EDLC) behavior. As the separation gap expands from 86 to 153 μm, the current density obviously diminishes at the same scan rate, indicating a reduction in capacitance, which is consistent with the classical capacitor theory. This capacitive phenomenon and performance are further verified by the GCD results in Figure 5i–k, which correspond to Figure 5f–h. The GCD curves are almost triangular across current densities from 0.2 to 5 mA cm^–2^ and the charge–discharge period also decreases with the separation gap expanding. For a more precise illustration of the superiority, the comparative analysis of the capacitive performance is conducted with a 2D LIG parallel plate capacitor (LIG-PPC), which has the same fabrication area and separation gap as one LIG-MRSC sample (Figure S12). Figure 5l,m show the areal capacitances of LIG-MRSCs and LIG-PPCs calculated from CV and GCD results (Figures 5f–k and S13). The areal capacitances of LIG-MRSCs in the CV test averagely surpass LIG-PPCs by over 6.56 times with identical fabrication parameters and even higher in the GCD test. Furthermore, the LIG-MRSC with the separation gap of 86 μm maintains an over 12.116 and 18.912 mF cm^–2^ areal capacitance at a scan rate of 20 mV s^–1^ and a current density of 0.2 mA cm^–2^, respectively. Figures 5n, S14, and S15 illustrate the cycling stability of LIG-MRSCs. For the mentioned sample, it averagely keeps the capacitance retention of over 97.2% and the Coulombic efficiency of over 96.9% at a current density of 1 mA cm^–2^ during the 1000 GCD cycles. The GCD curves consistently maintain triangular shapes across various cycling periods.

The above EDX and XPS results show silicon and sodium besides carbon. The +4-valent silicon element is likely to exist in the form of oxide, so it is difficult to dissolve and ionize. In addition, from the EDX results, the silicon element is evenly attached to the LIG, so it does not seem to form unique microstructures for the active material to attach. Sodium-containing compounds may react with the electrolyte to release sodium ions, which may eventually slightly enhance the electrochemical performance given the very low sodium content.

The LIG-MRSCs discussed in this subsection represent only one example of typical energy storage applications for the proposed LIG micro-rolls. It keeps attractive capacitive performance without any additional modifications or loaded active substances. This indicates that LIG micro-rolls have the potential to benefit numerous electrochemical applications due to the space fold strategy, which inspires researchers to explore the possibility of various combinations of LIG micro-rolls and other materials.

## Conclusions

This study presents a laser-guided approach to thin film self-assembly within the LIG fabrication process. Effective surface modification and appropriate laser parameters are crucial for the release and self-assembly of LIG thin films. The thermally cured Methylcellulose serves as an interlayer, while the gas bubbles that evaporate beneath the polyimide (PI) layer create weak adhesion, facilitating easier release of the films.

The transient ultrahigh temperatures generated by the laser induce thermal stress, promoting the rolling of the thin films. We demonstrated that both laser power and scanning line spacing can effectively control the diameter of LIG micro-rolls. Our systematic examination revealed an optimal laser power range of 0.96–1.24 W, with the outer diameter decreasing from 582 (±80) to 428 (±45) μm as power increased. Other parameters included a scanning speed of 400 mm s^–1^ and a line spacing of 2 μm. Further investigation into line spacing identified a workable range of 2–5 μm. Here, the outer diameter increased from 438 (±38) to 620 (±87) μm as the spacing expanded from 2 to 5 μm, with the same scanning rate and power settings. Notably, the inner diameter of the LIG microroll approached zero under the conditions of 1.24 W laser power, 2 μm line spacing, and a 400 mm s^–1^ scanning rate. Traditional thin film self-assembly methods struggle to achieve microscale curvature radii with 25 μm thick films, which indicates the significant temperature gradient and thermal stress present during the laser scribing process.

Additionally, the rolling areas and orientations of the thin films can be precisely controlled through tailored laser scanning paths and sequences, allowing long ribbons to be rolled from specified directions. Double micro-rolls can be created by rolling two adjacent patterned films in opposite directions, while asymmetric micro-rolls can be produced using varied patterns and scanning paths. This laser strategy offers substantial design flexibility for the creation of 3D LIG micro-rolls.

Finally, our proposed LIG-PI-LIG micro-rolls have been applied as small-scale supercapacitors as an exemplary verification. The LIG-MRSCs can achieve an EDLC of up to 334.10 mF cm^–3^ with a tiny device volume of 1.27 mm^3^. The impressive capacitive behavior illustrates the benefits of multiscale 3D graphene structures. By integrating the laser-guided self-assembly process into LIG fabrication, a low-cost, single-step, and controllable method for producing LIG micro-rolls is established with inspiring potential in fields like biosensing, water remediation, energy storage, etc.

## Methods

### Sample Preparation

The sample preparation process includes cleaning the substrate, applying a Methylcellulose solution, attaching the polyimide (PI), and thermal curing. Initially, a soda-lime glass substrate was cleaned by soaking in acetone and ethanol, followed by 5 min of sonication and drying with compressed air. A 2 wt % methylcellulose solution was created by dissolving methylcellulose in deionized water, using an electronic balance (BSM-220.4, Zhuojing, China) for accurate measurement. This solution was then applied to the glass surface. A 25 μm thick commercial Kapton film was placed onto the methylcellulose solution using tweezers and gently pressed to ensure full contact. The sample was then cured on a hot plate at 80 °C for 20 min. During this curing process, the water evaporated, causing the Kapton film to become uneven. A specially designed scraper was employed to remove any trapped air bubbles. As gas bubbles continued to form, the sample was periodically squeezed every 5 min to maintain an even surface.

### Laser-Induced Pattern Definition

This step utilizes laser engineering, which operates without a mask. AutoCAD software was used to create 2D pattern designs for the laser scanning process. A commercial 1064 nm continuous-mode fiber laser marking machine was employed, using a high power setting of 8.8 W to ablate specific areas of the Kapton film and define the patterns. The scanning speed was set at 400 mm s^–1^, and each pattern was written 50 times.

### Laser-Guided Self-Assembly

In this phase, the same commercial 1064 nm continuous-mode fiber laser marking machine was used. Lower laser power was applied to carefully scan the isolated thin film line by line, facilitating peeling and rolling. For diameter adjustments at different laser powers, the power range was set between 0.86 and 1.24 W, with a scanning speed of 400 mm s^–1^ and a line spacing of 2 μm. For adjustments based on varying line spacings, the range was from 2 to 6 μm, maintaining a laser power of 1.24 W and a scanning speed of 400 mm s^–1^.

### SEM and EDX Characterization

SEM images and EDX mapping results were obtained using a field emission scanning electron microscope (JSM-7800F, JEOL, Japan).

### TEM and EDX Characterization

TEM images and EDX mapping were collected using a double spherical aberration-corrected transmission electron microscope (Titan Cubed Themis G2 300/Titan Cubed Themis G2 30).

### XPS Characterization

XPS was done with the Axis Ultra DLD from Kratos Analytical.

### Raman Characterization

Raman spectra were recorded using a 532 nm light source with a Renishaw inVia Qontor Spectrometer System (RENISHAW, UK).

### LIG-MRSCs and LIG-PPCs Fabrication

First, A 1064 nm laser with power below 0.86 W was used to trigger the formation of LIG in the top layer, establishing a planar electrical interconnection area. This area was pasted by copper foil tape to guide signal wires and link them to the electrochemical workstation (CHI760E, CH Instruments, US). Then, the device was flipped, and the boundary was completely ablated through a laser with power exceeding 1.24 W, defining the area to be rolled up. Subsequently, a laser with power between 0.86 and 1.24 W was scanned to create LIG electrodes and to roll up the overall structure simultaneously (Figures S11b,c and S12). After the laser scanning, a hollow PDMS cap was affixed to the substrate (Figure S11d), and 1 M H_2_SO_4_ electrolyte was introduced via a syringe. The device was put in a vacuum flask for 1 h to eliminate bubbles before the electrochemical test.

## Supporting Information Available

SEM images for cutting and peeling PI film; laser-scribed PI film attached to the soda-lime glass with a DIW interlayer; laser-scribed PI film attached to the soda-lime glass with an ethanol interlayer; laser-scribed PI tape attached to the soda-lime glass; SEM EDX mapping results for the micro- and submicron-scale structures on the LIG micro-rolls; XPS characterization for a LIG-microroll; a LIG microroll with fused silica substrate; the laser power curves for adjusting LIG microroll diameters; the damaged LIG microroll with a laser power of 1.34 W; the asymmetric patterns and asymmetric laser scanning paths of PI films; the fabrication of LIG/PI/LIG micro-rolls and assembly of supercapacitors; optical images for LIG/PI/LIG micro-rolls; CV and GCD curves of LIG-PPCs; the cycling stability of LIG-MRSCs and LIG-PPCs; and the cycling stability of LIG-MRSCs (PDF)
One-step laser-guided fabrication of 3D self-assembled graphene micro-rolls (MP4)
