# Supplementary material for: One-Step Laser-Guided Fabrication of 3D Self-Assembled Graphene Micro-Rolls
Source: ACS Nano. 2025 Feb 3;19(5):5769–80. doi: 10.1021/acsnano.4c17646 (PMC11823605; doi:10.1021/acsnano.4c17646)
Supplement: Supplementary file 1 — nn4c17646_si_001.pdf [file nn4c17646_si_001.pdf]

Supporting Information

One-Step Laser-Guided Fabrication of 3D Self-Assembled Graphene Micro-Rolls

Yi Chen,†,‡,⊥ Xupeng Lu,†,⊥ Ganggang Ma,¶ Minseong Kim,† Ruohan Yu,§ Haosong Zhong,† Yee Him Timothy Chan,† Min Tan,†,‡ Yang Liu,‖ and Mitch Guijun Li*,†,‡

†*Center for Smart Manufacturing, Division of Integrative Systems and Design, The Hong Kong University of Science and Technology, Clear Water Bay, Kowloon, Hong Kong SAR, 999077, China*

‡*State Key Laboratory of Advanced Displays and Optoelectronics Technologies, The Hong Kong University of Science and Technology, Clear Water Bay, Kowloon, Hong Kong SAR, China.*

¶*State Key Laboratory of Advanced Technology for Materials Synthesis and Processing, Wuhan University of Technology, Wuhan, 430070, China*

§*Wuhan University of Technology, The Sanya Science and Education Innovation Park, Sanya, 572000, China*

‖*Department of Applied Physics, Hong Kong Polytechnic University, Kowloon, Hong Kong SAR, China.*

⊥Equal Contribution

E-mail: mitchli@ust.hk

Contents

Supplementary Figures 3

Supplementary Videos...19

Supplementary Figures

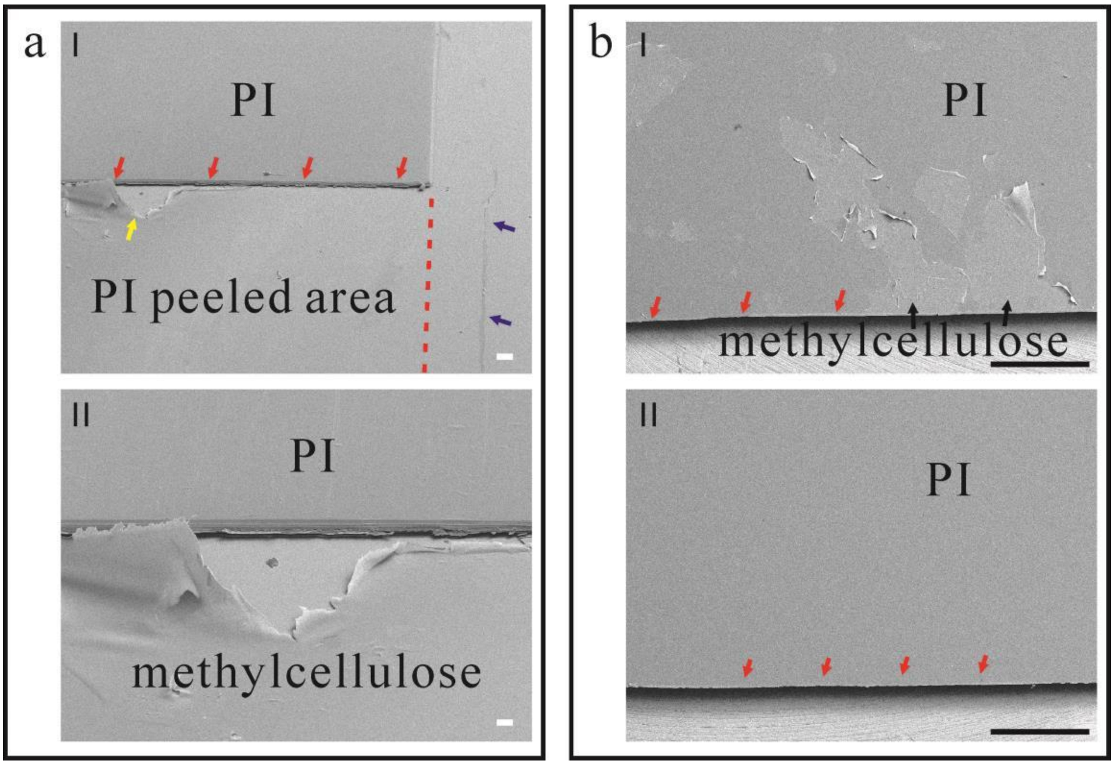

Supplementary Figure 1. The SEM images for cutting and peeling PI film. (a) A glass/methylcellulose/PI sample includes an area where the PI film is cut and peeled off (I), and an amplified figure of the methylcellulose broken and peeled area (II). The red arrows show the cutting edge of the PI film. The blue arrows show the boundary of the methylcellulose layer. The yellow arrow indicates the location of the broken and peeled methylcellulose layer. Scale bar, 100 μm. (b) The peeled PI film with some pieces of methylcellulose layer (I), and the peeled PI film without attached methylcellulose (II). The red arrows show the cutting edge of the PI film. The black arrows indicate the pieces of methylcellulose layer. Scale bar, 1 mm.

A glass/methylcellulose/PI sample was cut with a scalpel. Then the cut area was peeled off slowly with a tweezer. The blue arrows in Figure S1a-I show the boundary of the methylcellulose layer, which resulted from the squeezed methylcellulose solution with a scraper. The red dashed line indicates the original boundary of the peeled PI film. Figure S1a-II shows the broken and peeled methylcellulose location. It is obvious that

a nearly complete methylcellulose layer is attached to the glass substrate, while only a limited area is broken near the cutting edge.

Figure S1b-I shows the peeled PI film with some pieces of methylcellulose layer near the cutting edge. However, the methylcellulose pieces are minimal on the cutting edge of the peeled PI film. Figure Sb-II represents a more general condition: the peeled PI film is clean without methylcellulose.

The above results indicate a weak adhesion between the PI film and the methylcellulose layer.

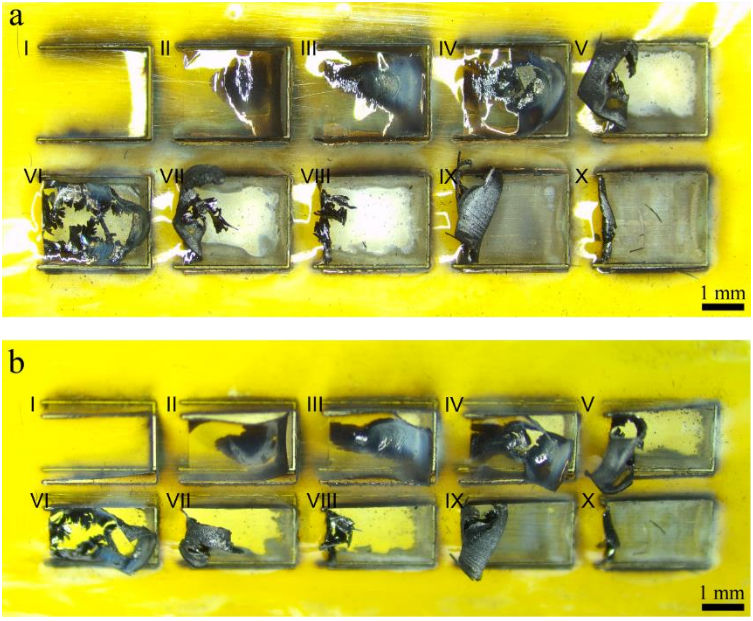

Supplementary Figure 2. The top view (a) and tilted view (45°) (b) of laser-scribed PI film attached to the soda-lime glass with a DIW interlayer, and the laser powers are (I) 0.46, (II) 0.56, (III) 0.66, (IV) 0.76, (V) 0.86, (VI) 0.96, (VII) 1.03, (VIII) 1.13, (IX) 1.24, (X) 1.34 W.

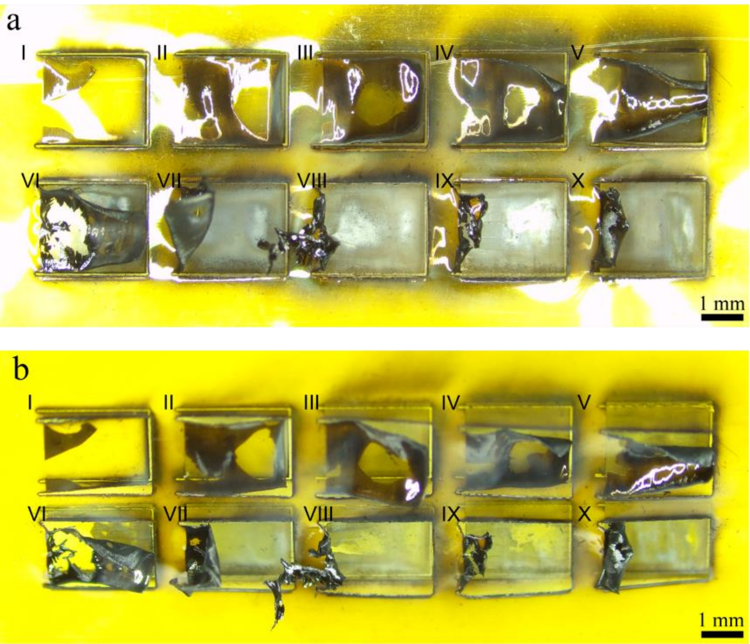

Supplementary Figure 3. The top view (a) and tilted view (45°) (b) of laser-scribed PI film attached to the soda-lime glass with an ethanol interlayer, and the laser powers are (I) 0.46, (II) 0.56, (III) 0.66, (IV) 0.76, (V) 0.86, (VI) 0.96, (VII) 1.03, (VIII) 1.13, (IX) 1.24, (X) 1.34 W.

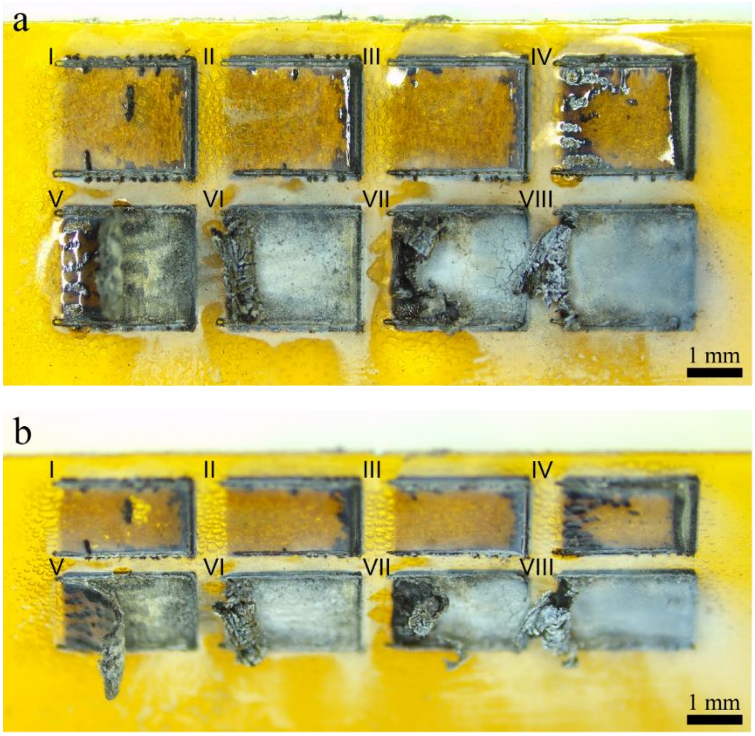

Supplementary Figure 4. The top view (a) and tilted view (45°) (b) of laser-scribed PI tape attached to the soda-lime glass, and the laser powers are (I) 1.24, (II) 1.34, (III) 1.44, (IV) 1.54, (V) 1.64, (VI) 1.74, (VII) 1.84, (VIII) 1.94 W.

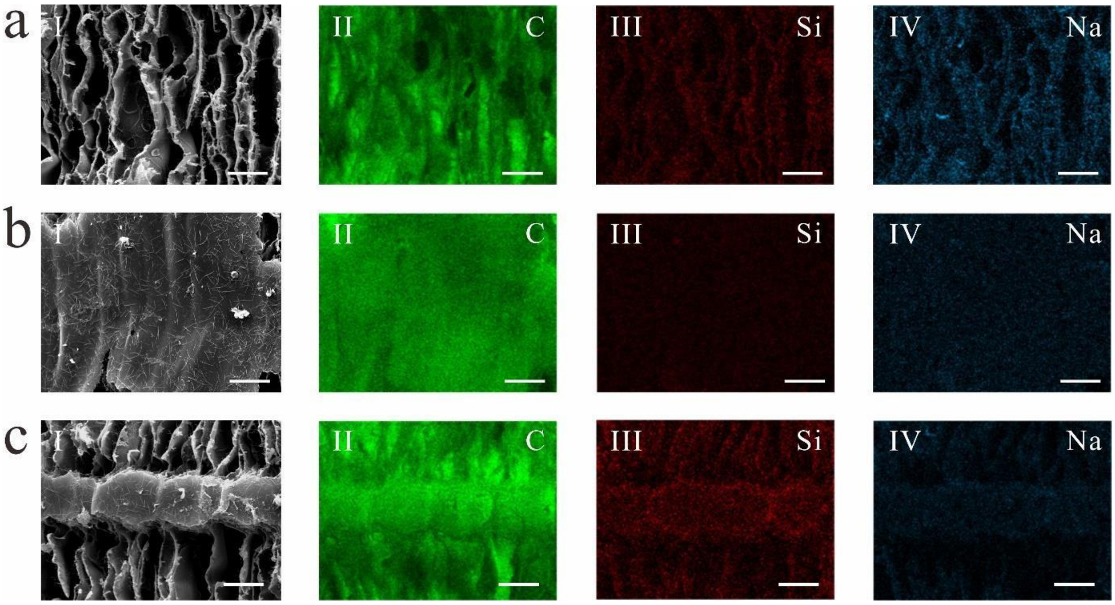

Supplementary Figure 5. SEM EDX mapping results for the micro- and submicron-scale structures on the LIG micro-rolls. (a) The flake structures (I) and EDX mapping results (II-IV). (b) The smooth structures (I) and EDX mapping results (II-IV). (c) The line structures (I) and EDX mapping results (II-IV). Scale bar, 10 μm.

The carbon, silicon, and sodium elements are evenly distributed on the flake, smooth, and line structures.

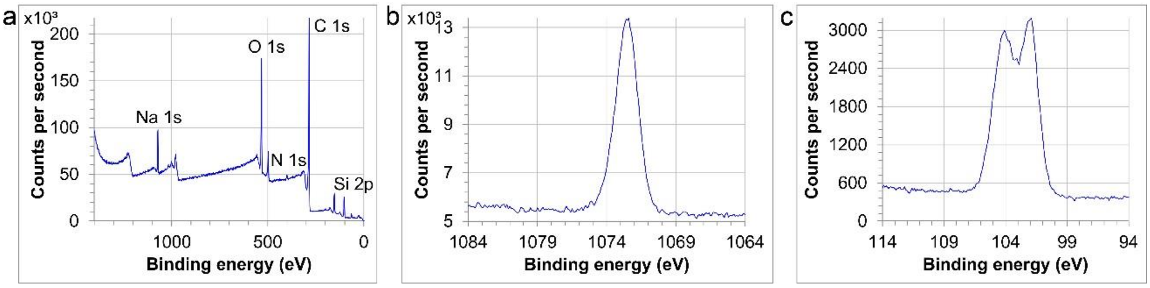

Supplementary Figure 6. (a) Typical XPS characterization for a LIG-micro-roll. (b) The XPS characterization for sodium element. (c) The XPS characterization for silicon element.

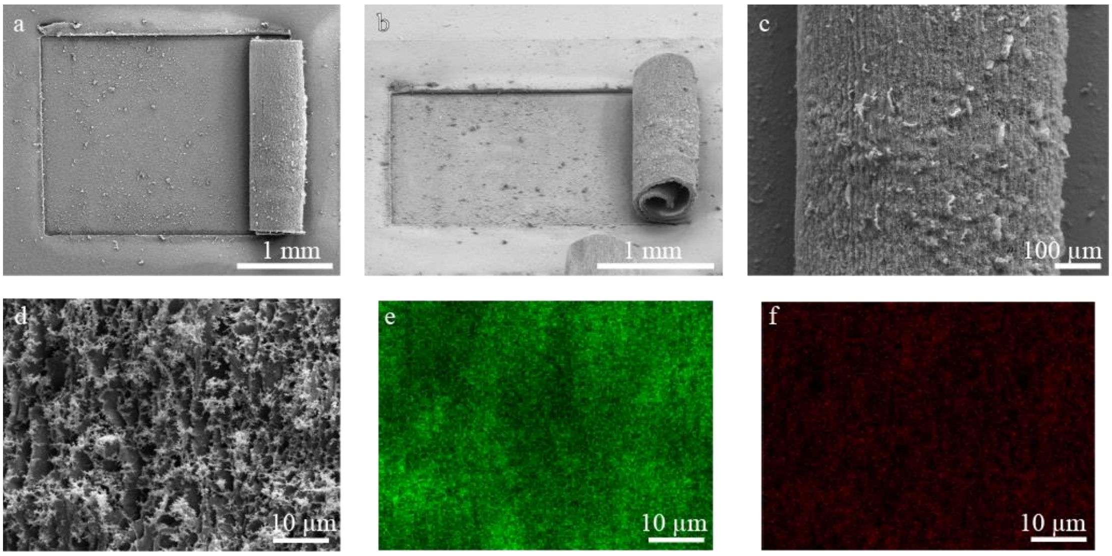

Supplementary Figure 7. The top view (a) and tilted view (55°) (b) of a LIG micro-roll with fused silica substrate. (c) An amplified area of the LIG micro-roll. (d) The EDX mapping area. (e) The EDX mapping result of carbon. (f) The EDX mapping result of silicon.

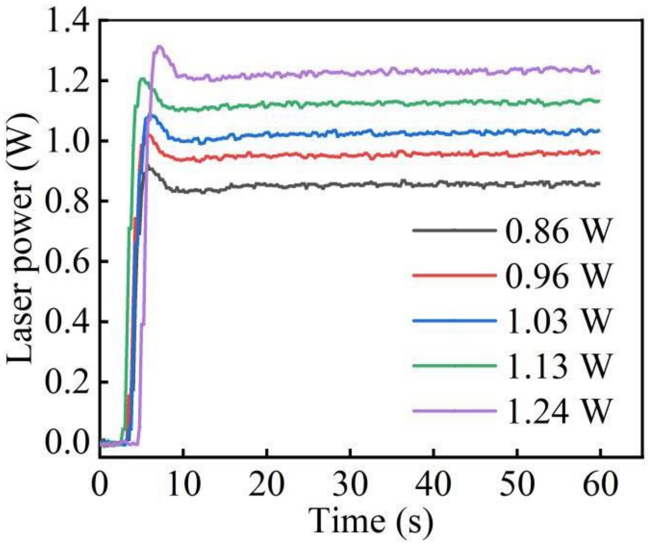

Supplementary Figure 8. The laser power curves for adjusting LIG micro-roll diameters.

The measurement time for each laser power is 1 minute. The laser power values are extracted after the values stabilize.

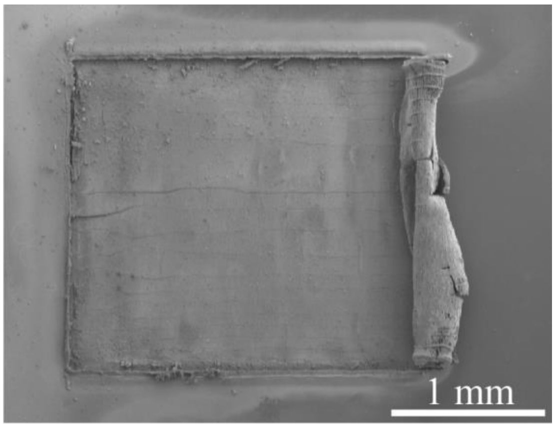

Supplementary Figure 9. The damaged LIG micro-roll with a laser power of 1.34 W.

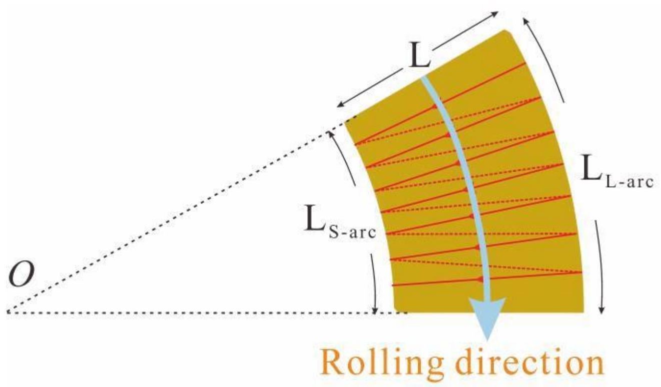

Supplementary Figure 10. The asymmetric patterns and asymmetric laser scanning paths of PI films.

Creating asymmetric micro-rolls can be broken down into two main steps: defining 2D asymmetric patterns and using laser guidance to roll these patterns in specific directions. This pattern consists of two arcs ($L_{L\text{-arc}}$ and $L_{S\text{-arc}}$) sharing the same center and angle, connected at both ends by straight lines (L). The intersection of the extended lines is precisely at the circle center (point O). The solid red lines indicate the laser scanning paths with power, and their extensions also intersect at the circle center, ensuring they are perpendicular to the arcs at their intersection points. The red dotted lines represent unpowered laser scanning paths that connect the solid red lines. The spacing between these scanning paths gradually reduces from the larger arc to the smaller one.

Initially, a 2D pattern is established through laser ablation. The laser then scans along the red lines to guide the asymmetric thin film to roll along both arcs and transition from one straight line to the other. The resulting micro-rolls exhibit different diameters at each end, reflecting the differences in arc lengths and laser scanning spacings.

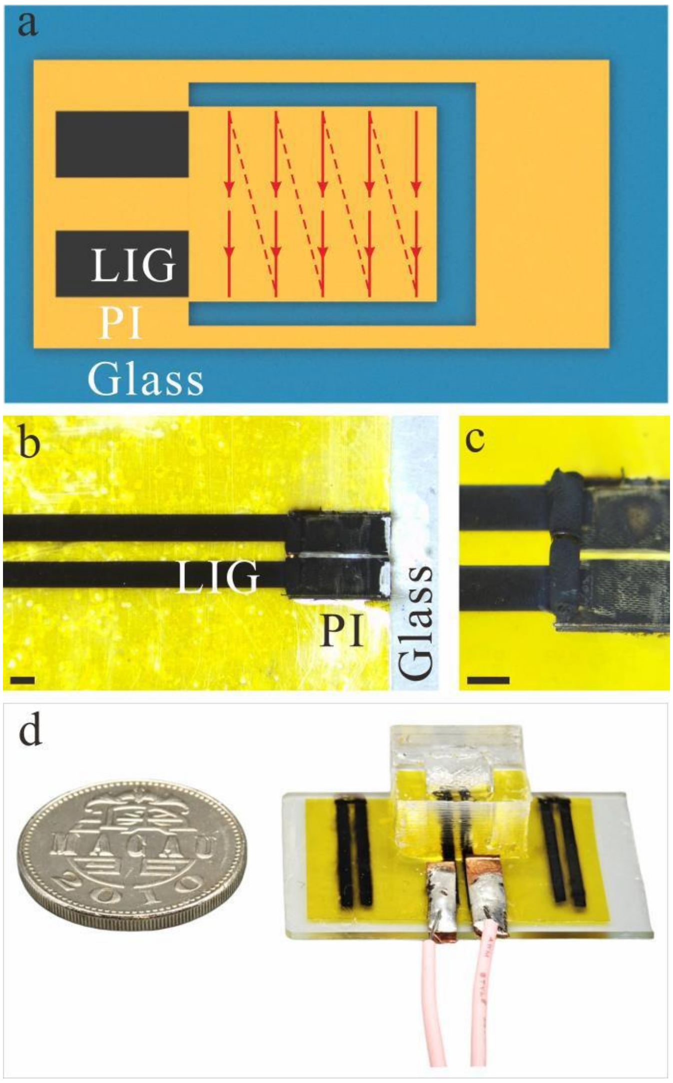

Supplementary Figure 11. The fabrication of LIG/PI/LIG micro-rolls and assembly of supercapacitors. (a) The illustration of laser scanning paths on the to-be-rolled area. The red solid lines and dashed lines represent the scanning paths with and without power, respectively. (b) A typical LIG/PI/LIG micro-roll. Scale bar, 1 mm. (c) A tilted view (45°) of the same LIG/PI/LIG micro-roll. Scale bar, 1 mm. (d) A typical supercapacitor, which shows a smaller size than 1 Pataca coin.

A PDMS chamber was placed on the LIG/PI/LIG micro-roll, and sulfuric acid (1 mol L^{-1}) was injected into it.

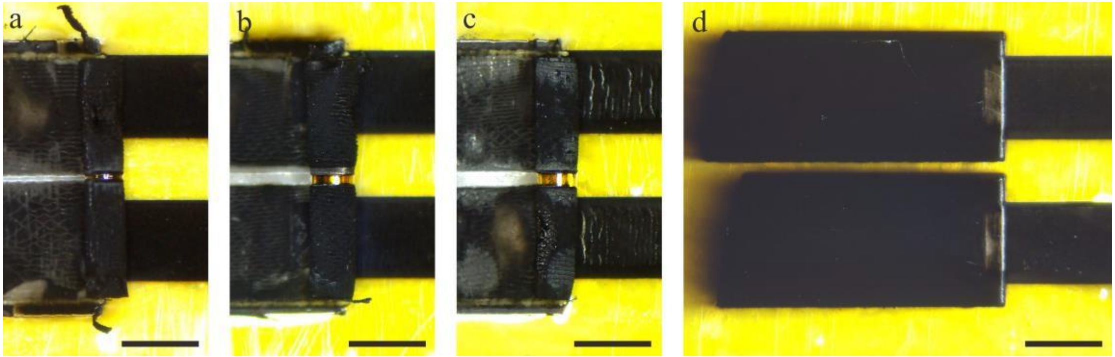

Supplementary Figure 12. Optical images for LIG/PI/LIG micro-rolls with PI widths of (a) 87 μm, (b) 120 μm, and (c) 153 μm. (d) A flatten LIG/PI/LIG of 153 μm. Scale bar, 1 mm.

For the LIG/PI/LIG micro-rolls, the black parts are LIG, and the yellow and transparent parts are PI.

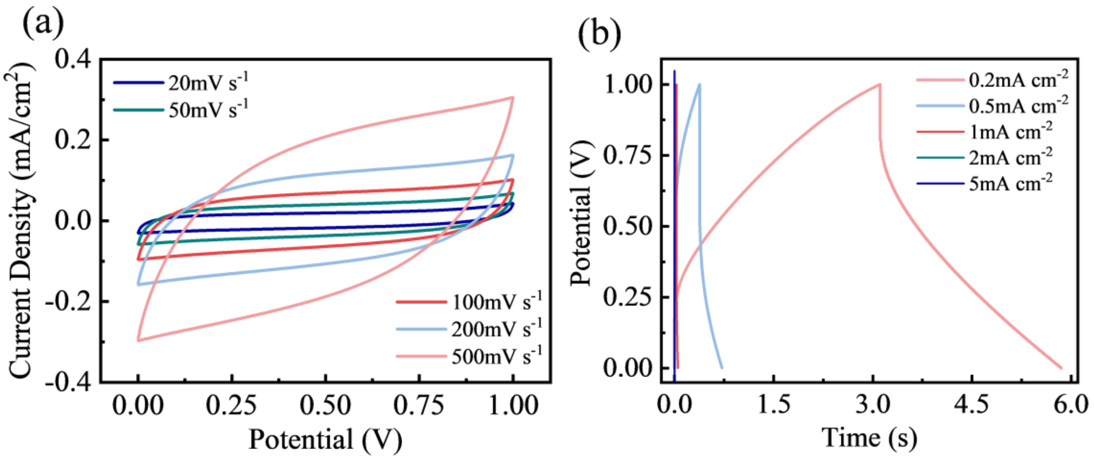

Supplementary Figure 13. (a) CV curves of LIG-PPCs across scan rates ranging from 20-500 mV s^{-1}. (b) GCD curves of LIG-PPCs across current densities ranging from 0.2-5 mA cm^{-2}.

LIG-PPCs illustrates a poor performance and inconclusive results in capacitance when the charge-discharge current density exceeds 1 mA cm^{-2}, primarily because the charge-discharge time closely aligns with the equipment's time resolution.

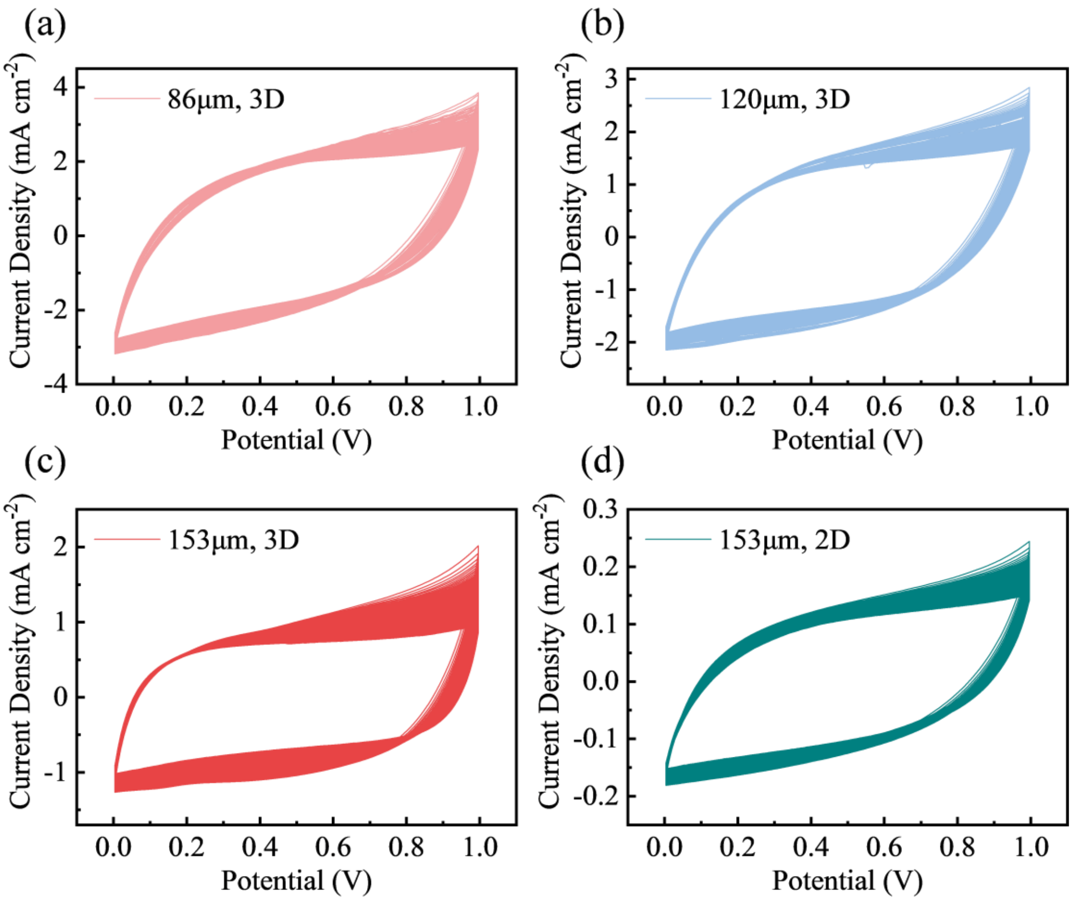

Supplementary Figure 14. The cycling stability of LIG-MRSCs and LIG-PPCs at a scan rate of 200 mV s^{-1} during the 1000 CV cycles. (a) LIG-MRSC, Gap=86 µm, (b) LIG-MRSC, Gap=120 µm, (c) LIG-MRSC, Gap=153 µm, (d) LIG-PPC, Gap=153 µm.

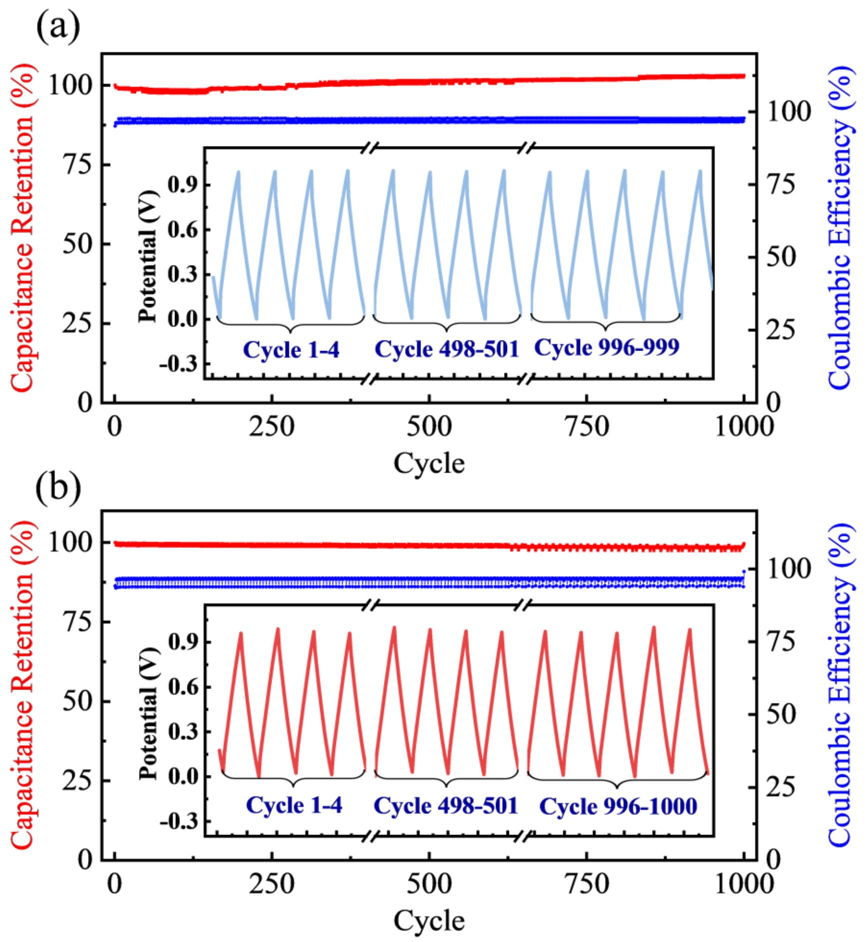

Supplementary Figure 15. The cycling stability of LIG-MRSCs at a current density of 1mA cm^{-2} during the 1000 GCD cycles. (a) Gap=120 μm, (b) Gap=153 μm.

Supplementary Videos

Video S1. Single-step fabrication of laser-induced graphene micro-rolls record

This video demonstrates the laser process on the PI/methylcellulose/glass sample. Initially, the laser outlines a pattern on the thin film. Next, it scans the patterned section line by line, transforming the PI into LIG while simultaneously releasing and rolling the LIG/PI film.
